# Comparison of Site-Specific Bone Mineral Densities between Endurance Runners and Sprinters in Adolescent Women

**DOI:** 10.3390/nu8120781

**Published:** 2016-11-30

**Authors:** Aoi Ikedo, Aya Ishibashi, Saori Matsumiya, Aya Kaizaki, Kumiko Ebi, Satoshi Fujita

**Affiliations:** 1 Graduate School of Sport and Health Science, Ritsumeikan University, Kusatsu 525-8577, Japan; gr0167si@ed.ritsumei.ac.jp (A.I.), gr0167kx@ed.ritsumei.ac.jp (A.I.), saori.m0824@gmail.com (S.M.), ab@fc.ritsumei.ac.jp (K.E.); 2 Department of Sports Science, Japan Institute of Sports Science, Nishigaoka, Kitaku, Tokyo 115-0056, Japan; 3 Department of Food Science and Nutrition, Mukogawa Women’s University, Nishinomiya 663-8558, Japan; 4 Research Organization of Science and Technology, Ritsumeikan University, Kusatsu 525-8577, Japan; kaizaki@fc.ritsumei.ac.jp

**Keywords:** adolescent, sprinters, endurance runners, bone mineral density, fat-free mass, nutrition

## Abstract

We aimed to compare site-specific bone mineral densities (BMDs) between adolescent endurance runners and sprinters and examine the relationship of fat-free mass (FFM) and nutrient intake on BMD. In this cross-sectional study, 37 adolescent female endurance runners and sprinters (16.1 ± 0.8 years) were recruited. BMD and FFM were assessed by dual-energy X-ray absorptiometry. Nutrient intake and menstrual state were evaluated by questionnaires. After adjusting for covariates, spine and total bone less head (TBLH) BMDs were significantly higher in sprinters than endurance runners (TBLH, 1.02 ± 0.05 vs. 0.98 ± 0.06 g/cm^2^; spine, 0.99 ± 0.06 vs. 0.94 ± 0.06 g/cm^2^; *p* < 0.05). There was no significant difference between groups in other sites. The rate of menstrual abnormality was higher in endurance runners compared with sprinters (56.3% vs. 23.8%; *p* < 0.05). FFM was a significant covariate for BMD on all sites except the spine (*p* < 0.05). Dietary intake of vitamin D was identified as a significant covariate only for pelvic BMD (*p* < 0.05). The BMDs of different sites among endurance runners and sprinters were strongly related to FFM. However, the association of FFM with spine BMD cannot be explained by FFM alone. Other factors, including nutrition and/or mechanical loading, may affect the spine BMD.

## 1. Introduction

Weight-bearing exercise has positive effects on bone metabolism across the age spectrum [1]. Adolescence is a critical time for bone mineral accrual [2]. Exercises that generate relatively high intensity loading forces enhance bone mineral accretion in adolescents [1]. Thus, adolescent athletes typically have higher bone mass compared with their nonathletic peers [3].

Endurance running has been associated with reduced risks for hypertension, hyperlipidemia, and diabetes [4]. Furthermore, regular running has been reported to reduce proportions of all-cause mortality and disability [5]. However, a subset of adolescent athletes may have impaired bone health [6,7]. Although endurance running is weight-bearing exercise, it has been associated with negative effects on bone in some populations, as indicated by reduced spine bone mineral density (BMD) in endurance runners [8,9]. In contrast, although both sprinters and endurance runners mainly use the lower limbs during exercise, sprinters demonstrate a higher BMD than endurance runners. The reason for a lower BMD in adolescent female endurance runners may be that this subject group has a greater running distance to cover, higher rate of menstrual irregularities, lower body mass index (BMI), and lower lean tissue mass [6] than sprinters of the same age group. Kusy et al. reported that sprinters in the masters age category have a higher BMD as well as bone mineral content (BMC) at the leg, hip, lumbar spine, and trunk than endurance athletes [10], whereas Bennell et al. reported that differences in the BMD between sprinters and endurance runners (17–26 years) exist only in the lumbar spine [11].

So far, the effect of the ground reaction force has been considered the most significant contributing factor for bone formation [11,12]. However, based on previous studies [10,11], the differences in BMD between sprinters and endurance runners could not be explained solely by the effect of the ground reaction force. Furthermore, although generally higher muscle mass and optimal nutrition is related with increased BMD, the effects of muscle mass and nutrition on BMD among endurance runners and sprinters have not been explored. Recent studies have only focused on BMD in endurance runners [6,7,8,9,13]. Clarifying the differences in site-specific BMDs between sprinters and endurance runners may reveal specific factors contributing to BMD gain in sprinters and endurance runners.

The aim of the present study was to compare site-specific BMDs between female adolescent endurance runners and sprinters, and to examine the relationship of fat-free mass (FFM) and nutrient intake with the BMD of different sites.

## 2. Materials and Methods

### 2.1. Study Design and Recruitment

In this cross-sectional study, we recruited 37 high school track and field female athletes (16.1 ± 0.8 years old; competition history of 3.4 ± 1.9 years), including endurance runners (>800 m, *n* = 16) and sprinters (100–400 m, *n* = 21). Study investigators recruited participants from five high schools in the Kansai district of Japan. The study protocol was approved by the Ethics Committee for Human Experiments at Ritsumeikan University (BKC-IRB-2013-031), and was conducted in accordance with the Declaration of Helsinki. All subjects and legal guardians of subjects provided informed consent for participation in this study.

### 2.2. BMD and Body Composition

We measured the height, body weight, and BMI of each subject. The body mass, fat mass, percent body fat, FFM, and bone mass were evaluated by a dual-energy X-ray absorptiometry (DXA, Lunar Prodigy; GE Healthcare, Tokyo, Japan). During DXA measurements, subjects maintained a supine position. From total body scans, we used enCORE version 15 software (GE Medical Systems Lunar, Madison, WI, USA), which automated measurements of FFM and fat mass (arms, legs, torso, gynoid (gluteal area), and total body), BMD (total bone less head (TBLH), arms, spine, pelvis, and legs), and percent body fat. For screening of at-risk athletes at younger than 20 years for low BMD, TBLH BMD measurement is recommended [14].

### 2.3. Menstrual State and Stress Fracture History

Menstrual state and stress fracture history were evaluated using questionnaires. For the menstrual state, the age of menarche and characteristics of the menstrual cycle were evaluated. Cycle lengths longer than 45 or shorter than 21 days were considered abnormal [15]. Stress fracture history was defined as having received a diagnosis of stress fracture in a medical institution.

### 2.4. Food Frequency Questionnaire

A food frequency questionnaire based on the food group (FFQg) was used to estimate usual energy and nutrient intake in athletes. The FFQg estimated nutrient intake from the ingestion frequency and food intake during one week from the most recent 1–2 months [16].

### 2.5. Physical Activity and Running Distance

Physical activity was estimated from three-day physical activity records. Subjects were instructed to estimate the practice time in minutes.

Running distance was estimated as the mean running distance per one-week from two-week running distance records. Physical activity and running distance were analyzed from the recovered questionnaires (33/37 questionnaires were recovered).

### 2.6. Statistical Analysis

Statistical analyses were performed with SPSS software version 19.0 (IBM, Tokyo, Japan). All values are expressed as mean ± SD. The independent *t*-test was used to determine differences in physical characteristics, FFM, and BMD between endurance runners and sprinters. An analysis of covariance (ANCOVA) was performed to compare BMD between endurance runners and sprinters, adjusted for age, height, FFM, and fat mass (of total body, arms, torso, gynoid (the gluteal area), and legs), menstrual abnormality, menarche, stress fracture history, and nutrient intake. Those variables that have been reported as important determinant of BMD in previous studies were selected as independent variables [3,6,17,18]. In addition, in a previous study, calcium and vitamin D were chosen as nutrients important for bone health [3]. Of those two nutrients, vitamin D was chosen as covariate, since there was a significant correlation with BMD in the current study. A *p* value < 0.05 was considered statistically significant.

## 3. Results

### 3.1. Subject Characteristics

Table 1 shows the subject characteristics. Endurance runners had a significantly higher running volume than sprinters (*p* < 0.01). Endurance runners also demonstrated a higher incidence of menstrual abnormality (*p* < 0.01) than sprinters. Table 2 shows the physical activities of subjects. Duration of practice was not different between two groups. However, running distance was significantly higher in endurance runners compared with sprinters. Table 3 shows the daily energy and nutrient intake. There was no significant difference between any parameters among the two groups.

### 3.2. Comparison between Endurance Runners and Sprinters

Table 4 shows subject FFM and BMD values. Endurance runners had a significantly lower FFM in all sites—except for the torso—compared to sprinters. Endurance runners had significantly lower arm, pelvic, spine, and TBLH BMDs than sprinters. However, the leg BMD was not significantly different between endurance runners and sprinters.

**Figure 1:**
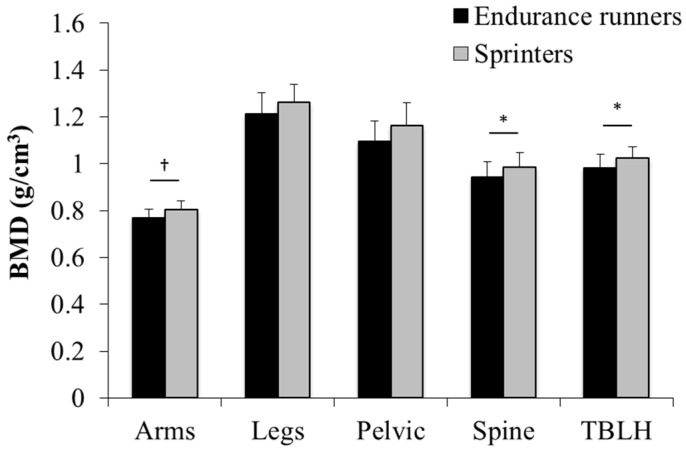
Comparison of adjusted BMD between endurance runners and sprinters. Endurance runners vs. sprinters; Spine: 0.94 ± 0.06 vs. 0.98 ± 0.06 g/cm^2^, TBLH: 0.98 ± 0.06 vs. 1.02 ± 0.05; *: *p* < 0.05, ^†^: *p* = 0.06.

In ANCOVA with adjustment for covariates such as age, height, FFM, fat mass, menstrual abnormality, menarche, stress fracture history, and vitamin D intake, the spine and TBLH BMDs remained significantly higher in sprinters than endurance runners (*p* < 0.05) (Figure 1). In contrast, there were no significant between-group differences in other sites.

### 3.3. Effect of Covariates on the BMD of Different Sites

In ANCOVA, FFM was a significant covariate for arms (*p* < 0.01), legs (*p* < 0.05), and pelvic (*p* < 0.05) BMD, and tended to be a covariate for TBLH BMD (*p* = 0.05) (Table 5). Additionally, vitamin D intake was identified as a significant covariate for arms (*p* < 0.05), pelvic (*p* < 0.01), and spine (*p* < 0.05) BMD, and tended to be a covariate for TBLH BMD (*p* = 0.05). Moreover, menarche was a significant covariate for arms BMD (*p* < 0.05).

## 4. Discussion

The purpose of this cross-sectional study was to compare BMDs of various sites and examine the association with different factors on the BMD among female high school track and field athletes. The main finding of our results was that endurance runners had significantly lower BMD in spine and TBLH as compared with sprinters, even after adjusting for covariates. In addition, vitamin D intake seems to have a site-specific association with arms, pelvic, and spine BMD. Furthermore, FFM was a significant covariate for most BMDs, with the exception of the spine.

### 4.1. The Difference between the BMD of Sprinters and Endurance Runners

When comparing the BMD of sprinters and endurance runners using a *t*-test, the BMDs of the arms, pelvis, spine, and TBLH in sprinters were significantly higher than those in endurance runners. However, after adjusting for covariates, between-group differences remained significant only for spine and TBLH BMDs.

In a previous study, ground reaction force with foot-strike during running was reported to be 1–2 times the body weight for low intensity forms of running (e.g., endurance) while it becomes 2–4 times the body weight for high intensity forms of running (e.g., sprint) [19]. According to the mechanostat theory, an increase in the bone mass is caused by larger bone deformation (e.g., high ground reaction force) which exceeds the normal strain for modeling [20]. However, the ground reaction force decreases as it is transmitted upward to the pelvis and spine from legs [13]. Since endurance runners experience smaller ground reaction force than sprinters, endurance runners may have less loading and deformation to spine bone when compared with sprinters with higher ground reaction forces. Additionally, in a previous study, weekly running volume was inversely correlated with lumbar spine BMD [21,22]. Greater running distance results in large energy expenditures, and one possible explanation for its effect on bone is via a potential catabolism when energy intake was insufficient, leading to low energy availability. Low energy availability has been shown to increase bone resorption and decrease bone formation, potentially mediated by reduced levels of insulin-like growth factor 1 or estradiol, resulting in low BMD [23,24]. Trabecular bone such as spine has been shown to be easily influenced by low energy availability [13]. Average running volumes of previously reported studies were 68.4 ± 12.1 km/week [21] and 32 ± 8 km/week [22] for endurance runners. Our current study participants exercised 58.5 ± 27.1 km/week among endurance runners and only 10.4 ± 5.3 km/week among sprinters, while their energy intake was identical between groups. Therefore, the low BMD of endurance runners may have been caused by both smaller mechanical loading as well as less energy availability as compared with sprinters.

Previous studies comparing the BMD of endurance runners and sprinters have often shown a difference in the leg BMD between the two groups [11,25]. However, this difference was not observed in the present study. The reason for this difference may be related to the subjects’ age and competition history. In a previous study, the subjects were over 17 years of age, and they had a competition history of over a decade [25]. In addition, in a previous study comparing the BMD of 13- to 18-year-old runners and non-runners, when separated by age, runners had significantly lower total body BMD compared with non-runners in the 17- to 18-year-old age group, but no difference was observed among groups of 13- to 16-years old [13]. The subjects in the present study had a mean age of 16.1 ± 0.8 years and mean competition history of 3.4 ± 1.9 years. Therefore, the lack of observed difference in leg BMD among long distance runners and sprinters may be caused by their age (bones being still in the growth stage) and relatively short competition history.

### 4.2. Relationship between Muscle Mass and BMD

After adjusting for age, height, FFM, fat-mass, menstrual abnormality, menarche, stress fracture history, and vitamin D intake, there were no significant group differences in the BMDs of the arms, legs, and pelvis. Among these confounding factors, FFM had the greater *F* value at each site. Thus, the FFM could have a strong influence on the BMDs among all sites. However, FFM was not found to be a significant covariate for spine BMD, while vitamin D intake was a significant covariate. Therefore, these results suggest that the spine might be more affected by nutrient factors such as vitamin D.

The close relationship between muscle mass and bone mass has been known for a long time [26]. In a previous study, sprinters were shown to have a higher FFM than endurance runners. Kusy and Zielinski [10] demonstrated that greater skeletal size allows exertion of larger muscle forces, supporting engagement in sprint disciplines, or forces exerted during sprinting induce skeletal adaptation and augment BMD. In addition, in a longitudinal study of 68 children (8 to 14 years), the maximal increase in lean body mass (LBM) occurred a several months before the maximal increase in BMC, indicating a close relationship between muscle and bone development [27]. These findings suggest that among adolescent female track and field athletes in their growth period, sprinters may have higher FFM and exercise intensity than endurance runners. Thus, in accordance with mechanostat theory, sprinters demonstrate higher BMDs than endurance runners.

### 4.3. Effect of Site-Specificity in Vitamin D

Vitamin D intake seems to have a site-specific relationship with arms (*p* < 0.01), pelvis (*p* < 0.05), and spine (*p* < 0.05) BMDs. A previous study using a vitamin D analogue indicated that the effect with vitamin D differs between cortical and trabecular bone. Takahashi et al. [28] concluded that vitamin D compounds might suppress receptor activator of nuclear factor-kappa B ligand (RANKL) activity in superficial osteoblastic cells of the trabecular bone. RANKL is an essential cytokine for activating osteoclast (increase in bone resorption). Therefore, habitual high vitamin D intake has a potential positive effect on pelvic and spine BMDs of trabecular bone. On the other hand, vitamin D intake was identified as a significant covariate for arms BMD. The bone of the arms consists mostly of cortical bone, since it is long bone. Thus, the aforementioned explanation for vitamin D and trabecular bone may seem inconsistent. However, FFM and menarche were demonstrated as significant covariates for arms. Since running puts minimal mechanical stress on arms, other variables such as FFM and nutrients may have had a larger influence. In the present study, the strongest covariate for arms BMD was FFM (*F* = 11.37, *p* < 0.01). However, since the results of the present study cannot explain the causal relationship, further study is warranted.

### 4.4. Study Limitations

This study included a relatively small sample size. Furthermore, the causal relationship cannot be determined by the current cross-sectional study without an inactive control group. Several parameters were not evaluated, such as bone metabolism markers, sex hormones (e.g., estrogen and progesterone), and reproductive maturation (such as tanner breast stage). Moreover, a previous study reported that subclinical ovulatory disturbance provides negative effect on bone [29]; however, the present study did not assess that. Low-dose oral contraceptives may impair the attainment of peak bone mass [30]. It should be noted, however, that the subjects of the present study were not taking oral contraceptives. In addition, FFQs for dietary assessment have been validated on collegiate woman, and not with the same age group of subjects in the current study. Accordingly, the dietitians used food samples to demonstrate the correct portion of specific foods. Future prospective studies are needed in other populations to determine variations, and intervention studies are warranted to determine the effects of FFM and vitamin D on site-specific BMDs.

## 5. Conclusions

We conclude that differences in the BMDs of different sites among endurance runners and sprinters were strongly affected by FFM. Furthermore, vitamin D intake also seems to have site-specific associations with BMDs. However, the relationship of FFM on spine BMD cannot be explained by FFM alone. Other variables, including nutrition (e.g., vitamin D) and/or mechanical loading may have been associated with spine BMD.

## Figures and Tables

**Table 1:** Subjects characteristics.

	Endurance Runners (*n* = 16)	Sprinters (*n* = 21)
Age	16.3 ± 0.8	16.0 ± 0.7
Menstrual abnormality (%)	56.3	23.8 **
Height (cm)	156.7 ±3.7	158.8 ± 4.5
Weight (kg)	47.6 ± 4.6	50.7 ± 5.3
BMI (kg/m^2^)	19.4 ± 1.5	20.1 ± 1.9
Fat mass (%)	19.9 ±4.6	19.5 ± 4.4

All values are mean ± SD; **: *p* < 0.01 vs. endurance runners.

**Table 2:** Physical activity.

	Endurance Runners (*n* = 14)	Sprinters (*n* = 21)
Practice time (min/day)	99.6 ± 38.7	109.7 ± 33.1
Running distance (km/week)	58.5 ± 27.1	10.4 ± 5.3 **

All values are mean ± SD; **: *p* < 0.01 vs. endurance runners.

**Table 3:** Energy and nutrient intake.

	Endurance Runners (*n* = 16)	Sprinters (*n* = 21)
Energy (kcal/day)	1927 ± 336	2099 ± 625
Protein (g/day)	70.0 ± 15.1	70.2 ± 20.5
Fat (g/day)	65.1 ± 18.1	78.1 ± 30.3
Carbohydrate (g/day)	258.5 ± 55.6	271.8 ± 78.8
Calcium (mg/day)	582 ± 205	595 ± 270
Magnesium (mg/day)	242 ± 52	232 ± 92
Phosphorus (mg/day)	1052 ± 251	1059 ± 345
Iron (mg/day)	7.5 ± 1.6	7.4 ± 3.1
Vitamin A (μg/day)	578 ± 161	553 ± 210
Vitamin D (μg/day)	6.4 ± 2.9	5.2 ± 2.9
Vitamin K (μg/day)	216 ± 65	182 ± 84
Vitamin B_1_ (mg/day)	0.97 ± 0.21	1.00 ± 0.35
Vitamin B_2_ (mg/day)	1.14 ± 0.32	1.21 ± 0.41
Vitamin B_6_ (mg/day)	1.09 ± 0.22	1.03 ± 0.40
Vitamin B_12_ (μg/day)	6.0 ± 2.3	5.4 ± 2.6
Vitamin C (mg/day)	104 ± 25	88 ± 43

All values are mean ± SD.

**Table 4:** FFM and BMD value among endurance runners and sprinters.

		Endurance Runners (*n* = 16)	Sprinters (*n* = 21)
FFM	Arms (kg)	3.2 ± 0.3	3.6 ± 0.4 **
	Legs (kg)	12.5 ± 1.3	13.7 ± 1.1 **
	Torso (kg)	17.0 ±1.7	17.8 ± 1.3
	Gynoid (kg)	5.4 ± 0.5	5.9 ± 0.5 **
	Total body (kg)	36.0 ± 3.2	38.3 ± 2.6 *
BMD	Arms (g/cm^2^)	0.767 ± 0.039	0.805 ± 0.038 **
	Legs (g/cm^2^)	1.211 ± 0.091	1.262 ± 0.077
	Pelvic (g/cm^2^)	1.097 ± 0.086	1.163 ± 0.099 *
	Spine (g/cm^2^)	0.942 ± 0.064	0.985 ± 0.062 *
	TBLH (g/cm^2^)	0.981 ± 0.061	1.023 ± 0.050 *

All values are mean ± SD; FFM: fat-free mass, BMD: bone mineral density, Gynoid: the gluteal area, TBLH: Total Bone Less Head; **: *p* < 0.01, *: *p* < 0.05 vs. endurance runners.

**Table 5:** Multivariable linear regression model on BMD of all subjects.

	Arms	Legs	Pelvic	Spine	TBLH
Age	0.48	1.74	2.61	0.06	0.48
Height	0.34	0.06	0.01	0.00	0.06
FFM (each site)	11.37 **	4.83 *	7.49 *	0.05	4.13 ^†^
Fat-mass (each site)	0.86	0.10	3.45	0.21	0.25
Menstrual abnormality	0.40	2.05	0.14	1.31	0.86
Menarche	6.13 *	1.78	0.17	1.04	1.61
Stress fracture history	2.00	0.97	0.18	0.88	1.29
Vitamin D intake	4.82 *	1.49	8.08 **	4.31 *	4.04 ^†^

All values are *F* values; **: *p* < 0.01, *: *p* < 0.05, ^†^: *p* = 0.05.

## References

[1] Kohrt W.M., Bloomfield S.A., Little K.D., Nelson M.E., Yingling V.R. (2004). American College of Sports Medicine Position Stand: Physical activity and bone health. Med. Sci. Sports Exerc..

[2] Gibbs J.C., Williams N.I., de Souza M.J. (2013). Prevalence of individual and combined components of the female athlete triad. Med. Sci. Sports Exerc..

[3] Mountjoy M., Sundgot-Borgen J., Burke L., Carter S., Constantini N., Lebrun C., Meyer N., Sherman R., Steffen K., Budgett R. (2014). The IOC consensus statement: Beyond the Female Athlete Triad—Relative Energy Deficiency in Sport (RED-S). Br. J. Sports Med..

[4] Williams P.T. (2009). Lower prevalence of hypertension, hypercholesterolemia, and diabetes in marathoners. Med. Sci. Sports Exerc..

[5] Chakravarty E.F., Hubert H.B., Lingala V.B., Fries J.F. (2008). Reduced disability and mortality among aging runners: A 21-year longitudinal study. Arch. Intern. Med..

[6] Barrack M.T., Rauh M.J., Nichols J.F. (2008). Prevalence of and traits associated with low BMD among female adolescent runners. Med. Sci. Sports Exerc..

[7] Tenforde A.S., Fredericson M., Sayres L.C., Cutti P., Sainani K.L. (2015). Identifying sex-specific risk factors for low bone mineral density in adolescent runners. Am. J. Sports Med..

[8] Bilanin J.E., Blanchard M.S., Russek-Cohen E. (1989). Lower vertebral bone density in male long distance runners. Med. Sci. Sports Exerc..

[9] Hind K., Truscott J.G., Evans J.A. (2006). Low lumbar spine bone mineral density in both male and female endurance runners. Bone.

[10] Kusy K., Zielinski J. (2015). Sprinters versus long-distance runners: how to grow old healthy. Exerc. Sport Sci. Rev..

[11] Bennell K.L., Malcolm S.A., Khan K.M., Thomas S.A., Reid S.J., Brukner P.D., Ebeling P.R., Wark J.D. (1997). Bone mass and bone turnover in power athletes, endurance athletes, and controls: A 12-month longitudinal study. Bone.

[12] Wosk J., Voloshin A. (1981). Wave attenuation in skeletons of young healthy persons. J. Biomech..

[13] Barrack M.T., Rauh M.J., Nichols J.F. (2010). Cross-sectional evidence of suppressed bone mineral accrual among female adolescent runners. J. Bone Miner. Res..

[14] Gordon C.M., Bachrach L.K., Carpenter T.O., Crabtree N., El-Hajj Fuleihan G., Kutilek S., Lorenc R.S., Tosi L.L., Ward K.A., Ward L.M. (2008). Dual energy X-ray absorptiometry interpretation and reporting in children and adolescents: The 2007 ISCD Pediatric Official Positions. J. Clin. Densitom..

[15] The American Congress of Obstetricians and Gynecologists (ACOG) (2006). Menstruation in girls and adolescents: Using the menstrual cycle as a vital sign. Obstet. Gynecol..

[16] Takahashi K., Yoshiyama Y., Kaimoto T., Kunii D., Komatsu T., Yamamoto S. (2001). Validation of a food frequency questionair based on food groups for estimating individual nutrient intake. Jpn. J. Nutr. Diet..

[17] Valimaki V.V., Alfthan H., Lehmuskallio E., Loyttyniemi E., Sahi T., Suominen H., Valimaki M.J. (2005). Risk factors for clinical stress fractures in male military recruits: A prospective cohort study. Bone.

[18] Heaney R.P., Abrams S., Dawson-Hughes B., Looker A., Marcus R., Matkovic V., Weaver C. (2000). Peak bone mass. Osteoporos. Int..

[19] Groothausen J., Siemer H., Kemper H.C.G., Twisk J., Welten D.C. (1997). Influence of peak strain on lumbar bone mineral density: An analysis of 15-year physical activity in young males and females. Pediatr. Exerc. Sci..

[20] Frost H.M. (1997). Why do marathon runners have less bone than weight lifters? A vital-biomechanical view and explanation. Bone.

[21] Winters K.M., Adams W.C., Meredith C.N., Loan M.D., Lasley B.L. (1996). Bone density and cyclic ovarian function in trained runners and active controls. Med. Sci. Sports Exerc..

[22] Burrows M., Nevill A.M., Bird S., Simpson D. (2003). Physiological factors associated with low bone mineral density in female endurance runners. Br. J. Sports Med..

[23] Ackerman K.E., Nazem T., Chapko D., Russell M., Mendes N., Taylor A.P., Bouxsein M.L., Misra M. (2011). Bone microarchitecture is impaired in adolescent amenorrheic athletes compared with eumenorrheic athletes and nonathletic controls. J. Clin. Endocrinol. Metab..

[24] Grinspoon S., Baum H., Lee K., Anderson E., Herzog D., Klibanski A. (1996). Effects of short-term recombinant human insulin-like growth factor I administration on bone turnover in osteopenic women with anorexia nervosa. J. Clin. Endocrinol. Metab..

[25] Magkos F., Yannakoulia M., Kavouras S.A., Sidossis L.S. (2007). The type and intensity of exercise have independent and additive effects on bone mineral density. Int. J. Sports Med..

[26] Doyle F., Brown J., Lachance C. (1970). Relation between bone mass and muscle weight. Lancet.

[27] Rauch F., Bailey D.A., Baxter-Jones A., Mirwald R., Faulkner R. (2004). The ‘muscle-bone unit’ during the pubertal growth spurt. Bone.

[28] Takahashi N., Udagawa N., Suda T. (2014). Vitamin D endocrine system and osteoclasts. BoneKEy Rep..

[29] Li D., Hitchcock C.L., Barr S.I., Yu T., Prior J.C. (2014). Negative spinal bone mineral density changes and subclinical ovulatory disturbances—Prospective data in healthy premenopausal women with regular menstrual cycles. Epidemiol. Rev..

[30] Hartard M., Kleinmond C., Wiseman M., Weissenbacher E.R., Felsenberg D., Erben R.G. (2007). Detrimental effect of oral contraceptives on parameters of bone mass and geometry in a cohort of 248 young women. Bone.

